# Joint Modeling of Longitudinal and Survival Data in Public Health and Biomedical Research: A Systematic Review

**DOI:** 10.3390/ijerph23040492

**Published:** 2026-04-13

**Authors:** Weize Wang, Zoran Bursac, Nan Hu

**Affiliations:** 1 Center for Research on U.S. Latino HIV/AIDS and Drug Abuse (CRUSADA), Robert Stempel College of Public Health and Social Work, Florida International University, Miami, FL 33199, USA; wwang033@fiu.edu; 2 Department of Biostatistics, Robert Stempel College of Public Health and Social Work, Florida International University, Miami, FL 33199, USA; zbursac@fiu.edu

**Keywords:** joint model, statistical methods, review, longitudinal and survival data, cardiovascular and cancer research, public health

## Abstract

**Highlights:**

**Public health relevance—How does this work relate to a public health issue?**

- Cardiovascular disease and cancer are leading causes of morbidity and mortality worldwide, often characterized by longitudinal risk factors and survival outcomes such as disease onset, progression, or death.
- This review examines recent advances in joint modeling methods developed across major public health areas, with emphasis on cardiovascular and cancer research, as well as applications in other chronic and complex diseases.

**Public health significance—Why is this work of significance to public health?**

- Joint modeling can reduce bias, improve estimation, and strengthen risk prediction when longitudinal and survival outcomes are related.
- This review summarizes recent methodological advances and shows different joint modeling sub-model approaches to address complex public health data structures.

**Public health implications—What are the key implications or messages for practitioners, policy makers and/or researchers in public health?**

- The review provides practical guidance for choosing longitudinal and survival sub-models based on the research question and data characteristics, particularly for cardiovascular and cancer applications.
- Better use of joint models can support more accurate risk assessment, clearer interpretation of the relationships between longitudinal biomarkers and survival outcomes, and more informed public health research and decision-making.

**Abstract:**

We conducted a PRISMA-guided systematic review to summarize recent methodological advances in joint modeling. A PubMed search for English-language, peer-reviewed, full-text available articles published between 1 January 2019 and 30 January 2025 was conducted using the keywords “joint model”, “joint modeling”, “longitudinal and survival”, “longitudinal and time-to-event”, and “public health”, resulting in 70 methodological studies from 793 records after screening. Original studies proposing methodological innovations in joint modeling were eligible, while clinical applications, reviews, comparative or predictive studies, and articles without full text were excluded. The reviewed methods introduced advances in both longitudinal and/or survival sub-models, including generalized linear mixed models, functional or latent class approaches, and flexible survival models, such as frailty, accelerated failure time, B-spline, and competing risks models. In total, 49% of the studies focused on longitudinal sub-model adaptations. This review is subject to limitations, including potential omission of relevant studies due to database, search term, and language restrictions. These developments have enhanced the flexibility of joint models for analyzing complex data structures, particularly in cardiovascular and oncology research, as well as broader public health applications. Despite these advances, challenges remain, including handling high-dimensional sparse data, reducing computational burden, and the lack of standardized evaluation metrics. This research received no external funding.

## 1. Introduction

In public health research, joint modeling of longitudinal and time-to-event data has been frequently used to analyze repeated measurements and their effects on a survival outcome simultaneously. Many public health studies collect biomarkers longitudinally while tracking the occurrence of health events, such as disease progression or death. Examples include monitoring CD4 counts in HIV surveillance, tracking cognitive function indicators in Alzheimer’s disease, and tracking the glomerular filtration rate for chronic kidney disease progression. Joint modeling helps reduce bias in estimation, improve estimation efficiency, and increase prediction accuracy [1]. Joint modeling takes informative dropouts and measurement errors of the repeated measurements into consideration. In addition, joint modeling provides insights into how the longitudinal trajectory of a measure is associated with the hazard of the outcome over time [2].

Before the development of joint modeling, an early approach for jointly analyzing longitudinal and time-to-event data was a two-stage model, where a linear mixed-effects model was first applied to a longitudinal covariate, and missing values were replaced by the predicted values from the model [3]. Then, a separate survival model was performed using the longitudinal covariate with both observed values and predicted values for the missingness of the first stage. However, there are many limitations to the two-stage models [4]. First, the estimation of the longitudinal parameters is biased in a two-stage model because it overlooks data truncations from events. The longitudinal model does not consider the survival event status during the estimation, and data will be missing after a subject experiences the event of interest. Second, the random effects and variance of the estimated parameters from the first stage are not integrated into the survival model in the second stage, resulting in biased estimates for the second stage. In addition, not all the available information in the data is used for estimation, so the efficiency of inference is limited [4].

To reduce the bias and improve the efficiency in estimation for longitudinal and time-to-event data, joint modeling was first introduced and applied in HIV studies by developing a joint maximum likelihood for the longitudinal and survival process simultaneously [5,6,7]. Longitudinal CD4 counts and progression to AIDS or mortality for each participant were recorded. Measurement errors are usually present in CD4 counts and viral load measures from laboratory errors and biological variability. Another aspect of the HIV longitudinal data is informative dropouts, where the slope of the CD4 count’s decline is directly associated with the risk of the outcome and non-random dropout [6]. Researchers then applied joint modeling to epidemic and clinical studies on primary biliary cirrhosis, end-stage renal disease, cancer, cognitive function, Huntington’s disease, Alzheimer’s disease, etc., where the study focus was the relationship between longitudinal biomarker measurements and patient survival.

In the past two decades, various expansions in statistical methods have been developed for joint modeling. Most of the work has been done based on the common approach of a joint model, which is composed of two sub-models, a longitudinal model and a survival sub-model, while the two sub-models are linked through shared random effects. The developed methods have employed adaptations in the longitudinal or the survival sub-models, or both. For example, nonlinear, generalized linear mixed modeling, latent class modeling, and B-splines have been used in the longitudinal sub-model, while accelerated failure time (AFT), frailty, and competing risk models have been applied as the survival sub-model.

When building on these methodological advancements, it is also important to review the existing joint modeling methods and to identify the remaining gaps. Most published review papers on joint modeling are introductory [4,8,9,10], non-methodological [11], or focused on software computational approaches [12]. Few studies have focused on the recent methodological development of joint modeling within public health research.

In this work, we performed a systematic review focused on recent developments in joint modeling methodologies of longitudinal and survival outcomes in public health research. The primary aim of this review is to present, compare, and evaluate recent joint modeling approaches. We will provide summaries and recommendations to guide future methodological development and applied research of joint modeling. The methods synthesized are highly relevant to cardiovascular and cancer research, where repeated biomarker measurements and clinical follow-up data are commonly collected alongside major event outcomes such as recurrence, progression, and mortality. In Section 4, we specifically address how these recent methodological advances can be applied to cardiovascular and oncology studies and provide recommendations to guide both future methodological development and applied joint modeling research in these major disease domains. We note that studies on estimation algorithms have contributed essential advances to the joint modeling methodology. However, this review specifically focuses on the development of longitudinal and survival sub-model methods, and studies primarily addressing estimation procedures were not included.

## 2. Methods

### 2.1. Search Strategy

The review adhered to the Preferred Reporting Items for Systematic Reviews and Meta-Analyses (PRISMA) guidelines (Supplementary Materials) [13], ensuring transparency and reproducibility through the selection and reporting process. We conducted a comprehensive search in the electronic database PubMed to identify relevant published research articles. The search strategy includes the keywords “joint model”, “joint modeling”, “longitudinal and survival”, “longitudinal and time-to-event”, and “public health”. Filters were applied to restrict results to peer-reviewed journal articles, in English, excluding pre-prints, with full-text availability, published between 1 January 2019 and 30 January 2025. The date last searched was 30 January 2025. The search period (1 January 2021 to 30 January 2025) was chosen to focus on the most recent methodological developments in joint modeling, as earlier foundational work has been reviewed in the prior literature. The registration DOI of the review is 10.17605/OSF.IO/NKUZF.

### 2.2. Study Selection

**Figure 1:**
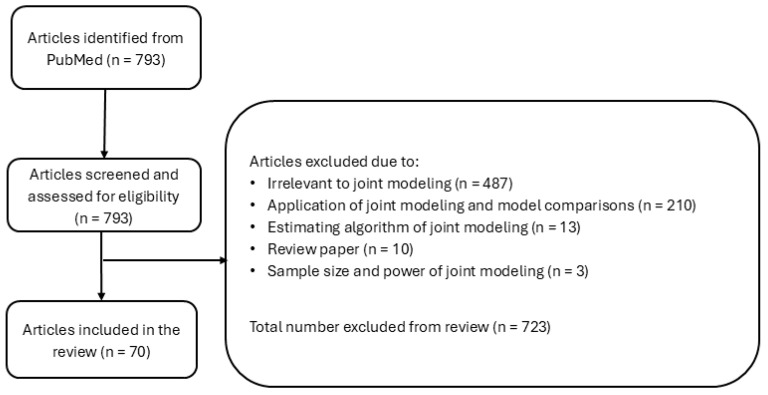
Flow chart of the literature search strategy (as of March 2026).

The initial database search identified 793 records (Figure 1). Title and abstract screening was then performed to evaluate relevance by a single reviewer to assess relevance to joint modeling methodology, with guidance and consultation from a senior reviewer to ensure consistency and resolve discrepancies. Studies were eligible for inclusion in the synthesis if they met the predefined eligibility criteria and contributed original methodological developments within joint modeling frameworks. Articles were excluded if their primary focus was clinical or epidemiological applications of joint modeling, comparative studies of existing joint models, predictive modeling only, reviews, studies not involving joint modeling methods, estimation algorithms, or sample size and power analysis for joint models. This screening resulted in the exclusion of 723 records. Full-text assessment of the remaining articles identified 70 studies that met the inclusion criteria. Throughout the selection process, uncertainties or eligibility questions were resolved through consultation with a senior reviewer.

### 2.3. Data Collection and Categorization

Data extraction was conducted by the same primary reviewer by reading through the methods sections of the selected articles, with supervision from a senior reviewer to ensure consistency and resolve discrepancies. Extracted data focused on the developments of methodological contributions, and included studies were classified according to novel developments in the longitudinal sub-model, the survival sub-model, or both within joint modeling frameworks. The results were summarized using structured tables presenting joint model characteristics and extensions, as well as application contexts. A qualitative narrative synthesis was conducted to compare methodological developments, identify common themes, and highlight remaining gaps in the literature. No quantitative data transformation or imputation was performed, as the synthesis emphasized methodological features rather than effect estimates. No automated data extraction tools were used, and study investigators were not contacted for additional information.

## 3. Review of Joint Modeling Methods

### 3.1. Standard Formation

The standard formation of the longitudinal sub-model is a linear mixed-effects model (LMM). Considering a sample of subjects followed over certain periods, let $Y_{i}(t)$ be a continuous outcome measured at time t for the ith subject, where i=1,…,n. The LMM has the form given in Equation (1):

$$Y_{i}\left(t\right)={X_{i}\left(t\right)}^{T}\beta +{Z_{i}\left(t\right)}^{T}u_{i}+\varepsilon_{i}\left(t\right)\;\;\;\;\;\;\;\;\;\;\;\;\;\;\;\;\;=m_{i}\left(t\right)+\varepsilon_{i}\left(t\right)$$

$$m_{i}\left(t\right)=X_{i}\left(t\right)\beta +Z_{i}\left(t\right)u_{i}$$

(1) $$u_{i}\sim N\left(0,\;\Sigma \right),{\;\varepsilon }_{i}\sim N\left(0,\;\sigma^{2}I\right)$$

where $X_{i}\left(t\right)$ and $Z_{i}\left(t\right)$ are the design matrices, with fixed effects, β, and random effects, $u_{i}$, respectively. $m_{i}\left(t\right)$ is a vector of the true value of the longitudinal outcome at time t. The random effects, $u_{i}$, are assumed to be distributed normally with a mean of 0 and a covariance matrix, Σ. $\varepsilon_{i}$ is the measurement error, and we assume that it follows a normal distribution with mean 0 and variance $\sigma^{2}$, independent across subjects. From the literature search, among the 70 studies on joint modeling methods in the past five years, about half of them (n = 34, 49%) focused on adaptations on the longitudinal sub-model. While using a standard Cox proportional odds model, the studies applied adaptations in the model for $Y_{i}\left(t\right)$. The survival sub-model of the joint model is meant to quantify the association between the longitudinal variable, $m_{i}\left(t\right)$, and the risk for a survival outcome. The Cox proportional hazards model is frequently used as the survival sub-model of a joint model:

(2) $$h_{i}\left(t\right)=h_{0}\left(t\right)\times \exp\left\{\gamma^{T}w_{i}+f(M_{i}\left(t\right))\right\},\;t>0$$

where ${\;h}_{i}(t)\;$ is the hazard of the survival outcome at time t for subject i, $h_{0}\left(t\right)\;$ is the baseline hazard function, $w_{i}\;$ is a vector of baseline or time-invariant characteristics, and γ is a vector of corresponding regression coefficients. Let $M_{i}\left(t\right)$ be the full history of the true values of the longitudinal variable up to time t, and $f\left(M_{i}\left(t\right)\right)$ is a function of the full history of the true values of the longitudinal variable.

### 3.2. Longitudinal Sub-Models

As we just mentioned, the standard formation of a joint model has two sub-models, the longitudinal and survival sub-models, and many expansions have focused on the longitudinal sub-models, with development for univariate and multivariate longitudinal outcomes. Methods that have been utilized are linear mixed-effects modeling (LMM), generalized linear mixed-effects modeling (GLMMIX), latent class modeling, models with latent variables, principal component analysis (PCA), nonlinear modeling, spatial–temporal modeling, and linear models with functional predictors. In the next section, we will discuss these modeling methods for the longitudinal sub-model of joint modeling.

#### 3.2.1. Univariate Longitudinal Sub-Models

- Linear mixed-effects sub-models

Because a standard joint model includes a linear mixed-effects sub-model, the expansion of joint modeling methods using the linear mixed-effects model usually emphasizes the longitudinal sub-model, particularly when dealing with multivariate longitudinal outcomes with more complexity. Nevertheless, one extension of joint modeling methods focusing on the univariate linear mixed-effects sub-model was to quantify the causal mediation effect of treatment between a longitudinal biomarker and the time-to-event outcome [14] (Table A1). In this study, the repeatedly measured biomarker trajectory was used as the mediator between the treatment/exposure and the survival outcome. Within the joint modeling framework, the total treatment effect on survival was decomposed into a natural direct effect and a natural indirect effect through the longitudinal mediator trajectory. These effects were under the sequential ignorability assumption, including correct temporal ordering and no unmeasured confounding of the mediation relationships. Further, robust linear mixed-effects modeling assumes that both the residuals and random effects follow a t-distribution instead of the standard normal distribution; by allowing for time-varying degrees of freedom, it has been employed to handle outliers in medical data [15] (Table A1). The proposed model assigns heavier tails to the error distribution to manage isolated measurement errors, while a t-distribution is used for the random effects to accommodate outlying patients who do not fit the general population trends. This effectively reduces the influence of outliers, preventing them from biasing the population-level estimates or the association with survival risk. The degree-of-freedom parameter is not fixed and can be time-varying. Meanwhile, the linear mixed-effects sub-model was used to reconstruct unobserved baseline covariate information in left-truncated survival data [16] (Table A1). A linear mixed-effects model was fitted to predict the covariate at the true baseline time, and then, the predicted baseline value was included in the survival sub-model. These methods demonstrate the flexibility of the linear mixed-effects sub-model in joint modeling, enabling causal mediation analysis between biomarkers and time-to-event outcomes and providing solutions for handling outliers and left truncation in medical data.

- Generalized linear mixed-effects sub-models

The normality assumption is frequently violated by biomarkers and certain longitudinal measurements in public health data. To make statistical inferences from joint models less biased and to improve prediction accuracy, generalized linear mixed-effects modeling (GLMM) was adopted when the longitudinal measurements, $Y_{i}\left(t\right)$, were not normally distributed. For univariate longitudinal outcomes, quantile regression [17,18,19], ordinal logistic regression [20,21], negative binomial [22], zero-inflated Poisson regression [23,24,25], and zero-inflated Beta regression [26] were adopted and applied as an extension of the longitudinal sub-model in the joint modeling (Table A1). An alternative and more complex method for semicontinuous data with excess zeros is the marginalized two-part model [27,28], which handles informative right censoring and is more robust for mis-specified random effect structures. Furthermore, GLMMs with log links for Poisson and binomial distributions [29] have been tested (Table A1). These adaptations and expansions allow joint models to handle non-normal, discrete, or zero-inflated longitudinal outcomes, making them applicable to diverse real-world cardiovascular and cancer data.

- Latent class analysis

Latent class approaches have been adopted in joint modeling analysis when assuming there is a latent class of heterogeneous subpopulations in the study population [30,31,32] (Table A1). For a univariate longitudinal outcome, an extension of the latent class joint model is the joint mixture cure model, in which the longitudinal sub-model is a standard linear mixed-effects model conditional on the class membership from a logistic model [31]. Latent class joint modeling has also been used to quantify the association between longitudinal measurements and the time-to-event outcome within each latent class, allowing the relationship between biomarkers and survival to vary across sub-populations [30]. These methods incorporate unobserved heterogeneity in disease progression. However, using latent class analysis in the longitudinal sub-model cannot evaluate the association between the biomarkers and the time-to-event outcome directly, and it is also challenging to interpret the latent classes explicitly. Further, latent class analysis methods in joint modeling have also been used with data censored at detection limits [32]. In this particular study, the longitudinal biomarkers were assumed to be conditionally independent of the survival outcome given latent class membership and correctly specified covariates [32]. This assumption is specific to this formulation and does not apply universally to all latent class joint models, which may allow for more flexible dependence structures between longitudinal and survival processes. Nonlinear longitudinal sub-models are also used.

Different association structures linking the longitudinal and survival processes have been compared while using a univariate B-spline mixed-effects sub-model alongside a semiparametric survival sub-model [33,34,35] (Table A1), while the piecewise linear spline method has been adopted to account for time-varying effects of treatment on the survival outcome over time along with a parametric survival sub-model [36] (Table A1). Cubic B-splines have been employed to flexibly model the time-varying coefficient [37] and define the unknown starting time of the endpoints [35], assuming the monotonicity of the data. Furthermore, a linear mixed model with natural cubic splines with different association structures linked to the survival sub-model has been explored [33], while B-spline and LASSO have been used to model the time-varying coefficients as the longitudinal sub-model [38]. Using B-spline in the longitudinal sub-model incorporates three levels of data hierarchy and spatial and temporal random effects, allowing for estimation using biomarker trajectories both over time and across geographic locations [39] (Table A1). The proposed model provides the time-varying effects of multilevel risk factors. Besides splines, nonlinear longitudinal sub-models can be represented using the longitudinal mean as a parametric nonlinear curve over time, such as a three-parameter logistic or an exponential-type curve, with subject-specific random effects [40] (Table A1). Using nonlinear models in the longitudinal sub-model provides joint models the flexibility of handling dynamic biomarker trajectories and their associations with survival outcomes.

- Discrete-time state space model

The longitudinal sub-process used the current true biomarker value, which depends on past values, following an autoregressive structure [41] (Table A1). This dynamic approach captures how biomarkers evolve for each patient individually, allowing for more accurate tracking of biological changes over time. By modeling the true biomarker process separately from observations, it provides a reliable input for assessing how these biomarkers relate to the risk of future clinical events. The model specifically improves the classical linear Gaussian state space model to include survival data, integrating the survival process at a latent level and leveraging the Markovian assumption for longitudinal progression.

#### 3.2.2. Multivariate Longitudinal Sub-Models

- Linear mixed-effects sub-models

Similar to the univariate linear mixed-effects sub-model for longitudinal outcomes, the approach of mediation analysis within a joint modeling framework was expanded to a case with multivariate longitudinal biomarkers in [42] (Table A2). Mediation analysis integrates marginal structural models with joint modeling, allowing for estimation of the direct and indirect effects of treatment via multiple longitudinal mediators on the time-to-event outcome and accounting for time-varying confounding factors. The method also provides the ranks of the effect sizes of mediators. The mediation analysis methods in joint modeling have been applied in cardiovascular disease, AIDS, and liver cirrhosis studies to evaluate the direct and indirect treatment effects. Furthermore, a linear mixed-effects sub-model with a multivariate normal distribution for multiple longitudinal factors has been studied [43] (Table A2). The study introduces a joint model that incorporates a multivariate linear mixed model for longitudinal trait trajectories with a frailty Cox survival model for multiple time-to-event outcomes.

- Generalized linear mixed-effects sub-models

When the longitudinal outcomes are multivariate and not normally distributed, a general linear mixed-effects model with skewed-normal distribution [44,45]; linear mixed-effects and ordinal logistic modeling for continuous and ordinal data [46]; multivariate quantile modeling [47]; and bivariate binomial modeling [48] are adopted (Table A2). Another scenario in joint modeling is that the longitudinal data are left-censored at detection limits. A “two-regime model” was developed by assuming that some missing values followed the same distribution as the observed values, and the rest of the missing values did not follow any parametric distribution [49] (Table A2). An additional method for non-normal longitudinal data is the general multivariate piecewise mixed-effects model, which analyzes correlated longitudinal measurements with random change points [50] (Table A2). To capture the dependence across multiple longitudinal outcomes, researchers introduced copula modeling with Gaussian and t distribution [51] and a Gaussian copula method, which links the marginal models and induces multivariate correlation without relying on shared random effects [52] (Table A2). Each longitudinal outcome was modeled separately using a marginal specification approach, typically through standard Bayesian regression methods. This copula-based approach enables the modeling of complex interdependencies among outcomes while preserving marginal interpretability [52]. Furthermore, the joint modeling was expanded to complex survey data by incorporating the pseudolikelihood method with sampling weights in [53] (Table A2). They used influence function surrogates from the survival sub-model, which was fitted using imputed phase-one target and auxiliary variables. These surrogates were then used to calibrate phase-two sampling weights and estimate the variances of the model parameters.

- Latent class and latent variable analysis

When the longitudinal data are high-dimensional, a latent class joint model with three sub-models, including a multinomial logistic model for latent class membership, a linear mixed-effect model for the longitudinal biomarkers, and a Cox proportional hazards model for the survival outcome, can be developed for high-dimensional correlated data [54] (Table A2). Later, a flexible joint model can incorporate generic association features (GAFs) and latent class structures to reduce dimensionality [55] (Table A2). Subjects are assigned to latent subgroups with distinct association patterns between longitudinal features and survival outcomes.

To examine the asymptotic properties of the joint modeling with latent classes, researchers performed a simulation study to understand how the censoring rate, sample size, and normality of the parameter estimates affect the performance of latent class identification [56] (Table A2). The researchers suggested that a small sample size and heavy censoring generate greater bias and errors in parameter estimation using latent class joint modeling.

Latent structures have been adopted in joint modeling analysis when the longitudinal measurements are high-dimensional or multivariate. In clinical practice, the longitudinal biomarkers are usually multidimensional, and the measurements are dependent on each other and the time to the event of interest. To deal with this issue, a factor analysis model was used as the longitudinal sub-model to imply the latent factor scores of the longitudinal covariables to lower the dimensionality and analyze multiple longitudinal biomarkers at the same time [57] (Table A2). Further, the joint modeling framework was expanded via a multidimensional latent-trait linear mixed model (MLTLMM) with different latent variables [58] (Table A2). The MLTLMM was developed through a two-level linear mixed sub-model with the latent variables, while the proportional hazards model was the survival sub-model. The hidden Markov model for the longitudinal responses was incorporated into joint modeling, along with confirmatory factor analysis for the longitudinal measurements to explore the dynamic covariate effects in the longitudinal trajectory [59] (Table A2). Nevertheless, the method was limited to dealing with complete data, and missingness was not taken into consideration.

#### 3.2.3. Principal Component Analysis and Functional Predictors

Functional principal component analysis has been incorporated into joint modeling to reduce the dimensions of longitudinal biomarkers along with a standard Cox regression [60,61,62,63] (Table A1). The method is composed of two steps: (1) performing functional principal component analysis on multiple longitudinal outcomes and generating a score showing the changing features of the longitudinal trajectories for each participant; (2) using the score generated from the first step as a covariate in a Cox regression. This two-stage approach enables individualized, time-updated risk predictions while maintaining interpretability. In particular, one study proposed a dynamic prediction framework using features extracted from multiple longitudinal outcomes and time-to-event data, where multivariate functional principal component analysis was used to summarize multiple neurocognitive markers into subject-specific scores [61] (Table A2). In another study, multiple highly correlated biomarkers were modeled through a shared latent reduced-rank longitudinal principal component model, in which the common latent trajectory was an additive combination of an overall mean curve and several principal component curves, with these functions modeled flexibly by B-splines [62] (Table A2). This approach allowed correlated biomarkers from the same biological pathway to share a common subject-specific stochastic trend over time without requiring a prespecified parametric time trend. In a related but different approach, the longitudinal outcome was modeled with a time-invariant functional predictor using imaging data, and the functional predictor used in the survival sub-model could be the same as or different from that used in the longitudinal sub-model [64] (Table A1). Principal component analysis was extended with functional survival forests, which replace the Cox model with a nonparametric random survival forest [63]. The random survival forest model uses summary scores from multivariate functional principal component analysis to jointly summarize correlated longitudinal outcomes, allowing the model to capture nonlinear associations and interactions. The latter method is more robust to sparse and noisy data. Both methods improve the prediction of disease progression, with the first providing structured inference and the second having advantages in handling complicated data. When longitudinal multivariate data are sparse and irregular, a multivariate functional mixed model is implemented in joint modeling [65]. This method uses multivariate functional principal component analysis (MFPCA) to summarize these sparse, noisy, time-varying measurements into a small set of features that capture each subject’s health trajectory, which are linked to the survival (Cox proportional hazards) model for risk prediction [66] (Table A2).

### 3.3. Survival Sub-Models

While advancements in the joint modeling methods have been focused on the longitudinal sub-models, development has also been undertaken in the survival sub-models. Semiparametric, parametric, and non-parametric survival models have been incorporated as survival sub-models.

#### 3.3.1. Semiparametric

Other developments in joint modeling have concentrated on the survival sub-model. While the semiparametric Cox proportional hazards model is the standard form in joint modeling, semiparametric AFT [35] (Table A1), Cox proportional hazards with piecewise baseline hazards [41], and B-splines have been explored in recent studies [29] (Table A1). Joint modeling has been applied to left-truncated data, where some participants are not at risk of the survival outcome at the study entry, and the time origin of the event of interest is unknown. AFT sub-models have been used to deal with the left truncation issue. A joint model with a semiparametric AFT sub-model was developed [35]. The method introduces a curve registration approach that characterizes individual disease progression using longitudinal biomarker trajectories, assuming a common shape function across subjects and allowing for person-specific time warping through random registration functions. Along with a B-spline smooth function to model the trajectory of the longitudinal process, a fixed unknown coefficient function with added semiparametric AFT sub-models has also been used in this scenario.

To improve prediction performance using time-varying biomarkers for recurrence risk, the linear state space dynamic survival model incorporates flexible time-varying covariates and evolving biomarkers for personalized hazard estimation, using hidden states from a state space model [41] (Table A1). The state space survival model shows remarkable predictive performance, especially when biomarker measurements are sparse or unbalanced. Researchers have also enhanced the traditional Cox sub-model by allowing nonlinear effects to flexibly influence hazard risk using B-splines [29]. Instead of constraining the proportional hazards model to linear covariate effects, the method allows the smooth, nonlinear functions of covariates to influence the hazard rate, providing greater flexibility and potentially better model fit.

Further, Cox hazard frailty models have been incorporated as the survival sub-model in joint modeling to account for the natural heterogeneity among individuals, for left-censored data [67], interval-censored survival data [68], and if there are more than one level of clustering factors [69] (Table A1). The Cox hazard frailty models in joint modeling have also been developed for the unmeasured dependence between events [43] (Table A2). Furthermore, frailty has been added to competing risk sub-models in the joint model using cause-specific hazard models [70] (Table A1).

A function of longitudinal factors that relate to the hazard of the event has been added to the survival sub-model to obtain a marginal estimate for the overall treatment effect in clinical trials [71] (Table A1). Distinct functional forms of association structures that show how the longitudinal process relates to the hazard, including the current value, slope, and cumulative value based on the deviance information criterion (DIC), have been evaluated [33] (Table A1). In addition, B-splines have been employed in Cox proportional hazard regression to model time-varying coefficients in the survival sub-model for potential interactions and nonlinear effects [38] (Table A1). The proposed method also enables variable selection with a penalized likelihood framework of adaptive least absolute shrinkage and selection operator (LASSO), providing automated selection of relevant predictors, distinguishing variables with static (time-invariant) effects from those with dynamic (time-varying) influences on both traits. Furthermore, a functional predictor using a cubic B-spline was included in the Cox model [64], supporting flexible representation of nonlinear and high-dimensional effects in the survival risk. The model enables dynamic estimation of the risk of disease progression for each patient with Alzheimer’s disease, updating the survival curve whenever new longitudinal or functional data becomes available. It provides spontaneous risk assessment and facilitates the development of personalized clinical interventions. In another study, the survival sub-model used a piecewise constant baseline hazard [36] (Table A1). This method allowed for time-varying hazard ratios. The model enables dynamic estimation of the risk of disease progression for each patient whenever new longitudinal information becomes available.

To allow time-varying coefficients to estimate age-related effects on survival outcomes, a semiparametric Generalized Odds Rate (GOR) model with a flexible age-varying effect of longitudinal process via splines and a nonparametric baseline hazard was developed [37] (Table A1). Depending on the coefficient estimates of the chosen parameters, the GOR model can be simplified to the proportional hazards model or to the proportional odds model. This development of a survival sub-model addresses situations where the relationship between a covariate and survival risk changes over time, and neither the proportional hazards nor the proportional odds models can fully capture it.

#### 3.3.2. Parametric

In the parametric survival models, the assumption is that the distribution of the survival time is known, including exponential, Weibull, log-normal, log-logistic, generalized gamma, etc., sub-models. The parametric AFT model differs from the semiparametric AFT in terms of parameter estimation, in that maximum likelihood can be used for parametric parameter estimation, while rank-based estimation is conducted in semiparametric estimation [72].

Among the survival sub-models, Weibull and exponential models have been frequently adopted [17,24,40,46,48,51,73,74,75] (Table A1 and Table A2). Gompertz models with baseline hazard specifications have also been employed [48] (Table A2). One AFT survival sub-model included a piecewise linear function to estimate the dynamic hazard ratio of a time-to-event outcome [73] (Table A1). Furthermore, log-normal, gamma, and piecewise exponential survival sub-models in the joint modeling have been adopted to account for delayed entry [75,76] (Table A1).

Recent developments have also introduced flexible hierarchical joint modeling frameworks, allowing for the incorporation of subject-specific random effects and complex baseline hazard structures. A flexible hierarchical joint model has been applied to jointly model longitudinal biomarkers and survival outcomes to improve risk factor identification and dynamic prediction [77]. Most of the joint modeling methods have been developed to handle a single cause of failure. For multiple survival outcomes, the joint modeling method has been extended by including failure time sub-models with competing risks [21,60,76,78,79,80,81,82] (Table A1). The baseline hazard for the hazard function of a particular survival outcome was specified using B-splines in [81] and the Weibull distribution in [21] (Table A1). The cumulative density function and proportional sub-distribution hazard model have been employed as the parametric survival sub-models in many recent studies [60,80,82]. In addition, a piecewise-exponential model was also used for competing-risk outcomes [76], while flexible regression with normal and generalized t-distribution has been employed [79].

The survival sub-model has been expanded as a continuous-time multistate Markov model, where individuals transition between predefined states based on the longitudinal markers and possible progress to the survival outcomes [20,83] (Table A1). The baseline hazard is defined as baseline transition intensity and specified using Weibull and piecewise constant baseline functions.

Later, researchers compared different specifications for the baseline hazard function within a joint model framework: piecewise-constant hazard, Weibull AFT, and B-spline hazard [84] (Table A1). The piecewise-constant and B-spline hazard versions are semiparametric. The piecewise-constant pseudo-adaptive Gauss–Hermite model has the best performance according to modeling fit, bias, and computation time.

## 4. Discussion

This review paper summarizes the recent advancements in joint modeling methodologies for longitudinal and time-to-event data in public health and biomedical research. By jointly analyzing repeated measurements and survival outcomes, joint modeling offers significant advantages for improving estimation efficiency and reducing bias due to informative dropout and measurement error. Joint modeling, by simultaneously analyzing repeated measures and survival outcomes, offers significant advantages in reducing bias and improving estimation efficiency. These strengths are particularly relevant to cardiovascular and cancer research, where longitudinal biomarkers (e.g., blood pressure, lipid levels, inflammatory markers, imaging measures, tumor burden, or treatment response indicators) are commonly collected alongside survival outcomes such as myocardial infarction, stroke, recurrence, disease progression, and mortality. Cardiovascular diseases remain the leading cause of death worldwide, responsible for nearly 20 million deaths annually [85]. Similarly, cancer is one of the top global causes of death, contributing to millions of deaths each year as a leading noncommunicable disease [86]. Together, cardiovascular disease and cancer represent a major proportion of the global mortality burden, highlighting the value of joint modeling approaches for generating reliable and less biased estimates in cardiovascular and cancer research. The present study highlights how joint modeling has developed in the last few years, emphasizing methodological advances in various disease settings. Our findings show that joint modeling has rapidly evolved in recent years through methodological innovations for increasingly complex biomedical data structures, supporting more accurate prediction and inference in high-impact disease settings, such as cardiovascular disease and oncology.

### 4.1. Recommendations for Sub-Model Choices

The findings of this review provide recommendations on the selection of sub-models in joint modeling. Although the longitudinal and survival components are estimated jointly, the considerations guiding their specification often differ depending on the characteristics of the repeated measurements, the event process, and the primary research objective. In particular, the distributional form, dimensionality, and temporal structure of the longitudinal outcomes mostly determine the choice of the longitudinal sub-model, whereas the nature of the event process and assumptions regarding the hazard function guide the choice of the survival sub-model. Based on the methodological developments identified in this review, recommendations are presented separately for the longitudinal sub-model, the survival sub-model, and the association structure.

#### 4.1.1. Recommendations for the Longitudinal Sub-Model

Methodological developments in joint modeling have focused extensively on the specification of the longitudinal sub-model. In practice, the choice of longitudinal sub-model should primarily be guided by the distributional characteristics of the longitudinal outcome(s), the dimensionality of the measurements, the presence of heterogeneity, and the research objective.

For continuous longitudinal outcomes that generally follow a normal distribution, linear mixed-effects models remain the standard choice and the most widely used sub-model. Extensions such as robust linear mixed-effects models can provide improved robustness when the data contains outliers or follows heavy-tailed distributions. When the longitudinal outcome is non-Gaussian, generalized linear mixed-effects models provide flexibility for different outcome types, including binary, count, ordinal, or proportional. Examples include logistic models for binary outcomes, negative binomial or Poisson models for count data, and ordinal logistic models for ordered categorical outcomes. Quantile models can also be considered for skewed outcomes.

For longitudinal outcomes with excessive zeros or semicontinuous distributions, zero-inflated count models and two-part models combining logistic and continuous components are recommended. For proportion data bounded between 0 and 1, such as microbiome measures, beta regression models or zero-inflated beta models provide an appropriate distributional specification. When measurements are subject to detection limits, two-part joint models can be employed to account for left-censored observations. In situations where the longitudinal trajectories exhibit nonlinear patterns over time, nonlinear mixed-effects models, spline-based models (including B-splines or penalized splines), and piecewise mixed-effects models can capture complex temporal dynamics. Penalized approaches, such as B-spline models combined with LASSO regularization, can further improve variable selection and model flexibility when multiple covariates or time-varying effects are present.

When the study population presents substantial heterogeneity or different disease progression patterns, latent class models or latent variable approaches can be used to identify subgroups with different longitudinal trajectories. These methods are particularly useful in chronic diseases such as cancer, where patients may follow different progression patterns. In addition, functional data approaches, including functional principal component analysis (FPCA) and multivariate FPCA, can effectively summarize complex longitudinal trajectories, especially when multiple biomarkers are measured repeatedly over time. For high-dimensional or multivariate longitudinal outcomes, several modeling strategies are available. Multivariate mixed-effects models, functional mixed models, and copula-based models allow for the joint modeling of multiple correlated biomarkers. In addition, spatiotemporal multilevel models can capture spatial dependence when measurements are collected across geographical locations. Overall, careful alignment between the characteristics of the longitudinal data, including outcome type, distribution, dimensionality, and temporal pattern, and the choice of longitudinal sub-model, is important for valid inference in joint modeling.

#### 4.1.2. Recommendations for the Survival Sub-Model

The survival sub-models have also advanced significantly, with researchers increasingly looking for alternative methods to the standard Cox proportional hazards model. Recent studies have explored the use of semiparametric models, including accelerated failure time (AFT) models, frailty models, and competing risks models to better handle left-truncated data, informative censoring, and multivariate survival outcomes. For studies involving multiple event types or competing risks, competing risk models are recommended for different causes of failure. Furthermore, multi-state Markov models are helpful for modeling transitions among multiple disease states, such as disease progression, relapse, and mortality. These models are especially useful for chronic diseases in which patients may progress through several stages over time. When greater flexibility in modeling the baseline hazard is needed, piecewise constant hazard models or B-spline baseline hazard models can capture the complex hazard shapes. These methods allow the hazard function to vary over time. When the time-to-event distribution can be assumed, parametric survival models, such as AFT models based on Weibull, exponential, or Gompertz distributions, may be considered. Parametric models are especially useful in clinical trials or mechanistic studies when the underlying hazard can be reasonably assumed. When recurrent events or clustered survival outcomes are present, shared frailty models can account for unobserved heterogeneity and within-cluster correlation. These models are particularly useful in multicenter studies. Further, models with time-varying coefficients or functional predictors allow covariate effects to change over time, making them helpful when the proportional hazards assumption does not hold. More recently, survival sub-models with a machine learning approach, such as random survival forests, have also been explored in joint modeling for complex nonlinear relationships or high-dimensional predictors. Overall, the choice of survival sub-model should be guided by the characteristics of the time-to-event process, including the number of event types, whether having recurrent events or not, assumptions regarding the hazard function, clustering effects, or time-varying effects.

#### 4.1.3. Recommendations for the Association Structure

The choice of association structure should reflect both the biological context and the relationship between longitudinal biomarkers and the survival outcome. For biomarkers with a direct and consistent effect on the event, such as CD4 count in HIV/AIDS or serum creatinine in kidney disease, the shared parameter model is generally recommended, capturing risk through current value or slope components. In the meantime, for complex, multi-stage diseases like Alzheimer’s disease or cancer, latent class association structures are recommended to account for distinct patient characteristics with varying biomarker-survival relationships. When dealing with skewed or non-normally distributed biomarkers, such as viral loads or microbiomes, copula-based structures can be used to capture nonlinear dependencies. Finally, studies with many correlated biomarkers—functional or high-dimensional latent trait structures—can help with identifying disease progression. Selecting an association structure that aligns with the clinical and statistical context ensures more robust, interpretable, and biologically meaningful inferences.

Across the reviewed studies, estimation of the joint modeling used either likelihood-based or Bayesian methods. Because these models require integration over subject-specific random effects, many studies used numerical approximation techniques such as adaptive Gaussian quadrature or Laplace approximation. Bayesian approaches, often implemented through Markov chain Monte Carlo (MCMC), were more common in studies with more complex settings, such as nonlinear trajectories, multiple longitudinal outcomes, or high-dimensional biomarkers. Some recent studies also used approaches such as stochastic approximation or penalized estimation to reduce computational burden and improve flexibility. Overall, the computational strategy usually depended on how complex the longitudinal and survival sub-models were and how much data the model needed to handle. In practice, it is also important to consider how well a joint model fits the data and prediction accuracy, and the interpretability of the results for public health decisions. Model fit can be checked using measures such as −2 log likelihood, AIC or BIC, residual plots, and comparisons between observed and predicted longitudinal trajectories. In joint modeling, predictive performance is typically evaluated using dynamic prediction measures such as the time-dependent concordance index, time-dependent area under the curve (AUC), and Brier score or integrated Brier score, along with calibration plots to assess agreement between predicted and observed risks over time. At the same time, the interpretability of the results from joint modeling is also crucial for developing future clinical or public health interventions.

We did not perform simulation studies or include a real-data application to numerically compare the reviewed methods. As discussed above, different joint modeling approaches are designed for different scientific settings, data structures, and research objectives. Because of these differences, they are not directly comparable within a single unified simulation or example without substantially narrowing the scope. Moreover, using only one example might give the impression that the methods can be broadly ranked, which would be misleading given their context-specific purposes. Therefore, direct numerical comparison is beyond the scope of this review, and method selection should be guided by the specific research question and characteristics of the data.

Despite these advancements, several challenges remain in the joint modeling framework. For example, the handling of high-dimensional data is still an area of active research, as many biomarkers in clinical studies are measured over time and across multiple subjects. Methods like functional principal component analysis (FPCA) and latent class models have been proposed to reduce dimensionality and capture the essential features of longitudinal data, but these techniques require further refinement and extension to different diseases. Additionally, the incorporation of time-varying covariates into the survival sub-model introduces complexity in model interpretation and parameter estimation. Another challenge in joint modeling is the need for robust estimation techniques, especially when dealing with sparse or noisy data. Many of the joint models utilized Bayesian methods for estimation, but this approach can be computationally intensive, particularly for complex models with large datasets. As such, balancing computational efficiency with model complexity remains a critical consideration for future research.

In terms of clinical application of the joint modeling methods, joint models have been increasingly applied to a wide range of diseases, with particularly strong relevance to cardiovascular and cancer research. In cardiovascular studies, joint models have been widely applied to investigate the dynamic relationship between longitudinal risk factors, such as lipid profiles, blood pressure, and physical activity, and survival outcomes, including coronary heart disease and cardiovascular mortality [21,34,37,42,74]. These approaches are especially valuable for risk prediction and precision prevention, as they incorporate repeated biomarker measurements to capture temporal changes in disease risk. Given the global burden of cardiovascular disease [85], joint modeling provides a powerful framework for improving early detection, prognostic evaluation, and individualized intervention strategies. In cancer research, joint models have been applied to a variety of tumor types. Longitudinal outcomes such as tumor burden and progression-free survival were studied in non-small cell lung cancer [69]. Longitudinal quality-of-life measures and time to relapse, second primary cancer, or death were analyzed in breast cancer [36,52]. Tumor size and time to death were modeled jointly in glioblastoma [29]. Carcinoembryonic antigen (CEA) and time to death were used in colon cancer [84]. Tumor measurements and competing risks of death were investigated in melanoma [70]. In prostate cancer, longitudinal prostate-specific markers and time to death were jointly modeled to assess treatment effects [81], multiple biomarker trajectories, and the time to relapse of acute lymphocytic leukemia were also jointly modeled [47].

Beyond cardiovascular and cancer research, joint modeling has also been extensively developed and applied to other chronic and complex diseases. In neurological disorders such as Alzheimer’s disease [61,63,64,65], amyotrophic lateral sclerosis [56,58], Huntington’s disease [18], and other cognitive degenerative diseases [48,59], neurocognitive markers are typically modeled as longitudinal outcomes, while time for disease progression or death serves as the survival outcome. In HIV/AIDS research, joint modeling is commonly applied to study the relationship between longitudinal CD4 cell counts and time to clinical progression or death [14,19,20,23,24,25,33,51,82,83]. The method has also been extended for HIV vaccine studies, linking longitudinal immune response biomarkers with time to HIV infection [49]. Moreover, joint modeling methods have been widely applied to diabetes and kidney disease research. For diabetes, longitudinal risk factors include HbA1c, systolic blood pressure, weight, and height, time to type-1 diabetes [26,43,45,50], and antidiabetic treatment failure [16]. In kidney disease, the longitudinal glomerular filtration rate (GFR) is often analyzed jointly with time to end-stage renal disease [39], or time to transplant failure or death [60]. In cystic fibrosis, longitudinal lung function measures have been analyzed jointly with time to death or lung transplantation [30,75]. In pulmonary fibrosis, longitudinal molecular and clinical biomarkers have been modeled together with time to death or disease progression [54,57]. In addition, joint modeling has been used in cardiovascular disease research. Longitudinal lipid profiles and time to coronary heart disease have been analyzed to study cardiovascular risk progression [74]. Longitudinal health indicators and exercise measures have been linked with time to cardiovascular disease events or mortality [21,34,37,42]. Furthermore, joint modeling has been advanced by primary biliary cirrhosis research. Longitudinal biomarkers such as bilirubin, serum albumin, hepatomegaly, histologic stage of disease, alkaline levels, and time to mortality have been modeled simultaneously [15,38,44]. Other applications include inflammatory biomarkers with time to pneumonia [32,46,67]; repeated measures of a woman’s Prevotella abundances or cervical dilation and time to delivery [23,79]; longitudinal HCG trajectory; and time to miscarriage [40], and other longitudinal measurements with death in intensive care unit (ICU) settings [78,80]. Overall, the flexibility of joint models enables inferences of the longitudinal effects on survival outcomes, which is particularly critical in cardiovascular and cancer research, where disease progression is dynamic. Future work in joint modeling should focus on further improving computational algorithms, enabling more widespread use of these models in large-scale clinical studies. The development of user-friendly software and simulation tools will also be important to facilitate the employment of joint modeling methods for application.

### 4.2. Limitations

While this review provides a comprehensive summary of recent methodological developments in the joint modeling of longitudinal and survival data for public health research, especially for cardiovascular and cancer research, several limitations should be considered. First, the scope of recent methodological innovation in joint modeling is partly constrained by available published literature in the electronic database PubMed. Some articles may have been missed due to limitations in search terms or database indexation, especially for interdisciplinary work published outside traditional biostatistics journals. In addition, restricting inclusion to English-language articles may have excluded relevant research published in other languages. Most articles identified focus on modifications or extensions to established joint modeling frameworks, often targeting application areas of a specific disease. This may limit the generalizability of certain approaches to other domains or populations.

Second, the reviewed studies vary considerably in their methodological design and reporting practices. Many methodological papers rely primarily on simulation studies, while other studies also demonstrated the proposed methods using real data. These differences in study design, sample size, and application may affect the generalizability of the methods to other settings. The diversity of joint modeling methodological advances may result in inconsistent reporting quality and evaluation standards, which can complicate efforts to directly compare methods.

Moreover, there are several methodological limitations in the review process. First, the study did not formally examine the quality of reporting or risk of bias for included articles, as is standard for reviews of research articles. Second, the review of title and abstract screening was conducted by a single author under the guidance and supervision of a senior author, which may introduce potential selection bias. In summary, while substantial effort was applied to ensure a comprehensive review, both the included evidence and the review process are subject to limitations that should be considered when interpreting the findings and recommendations for future research.

## 5. Conclusions

The original joint models can estimate longitudinal effects and time to events simultaneously. Recent advancements in joint modeling have greatly enriched the existing JM methods for analyzing longitudinal and survival data in public health and biomedical research: (1) Joint modeling has been improved by reducing estimation errors, allowing time-varying coefficients, and including flexibility for different types of longitudinal covariates. (2) The recent advancements in joint modeling have significantly enriched the existing joint modeling methods for analyzing longitudinal and survival data in public health and biomedical research. (3) While challenges remain, particularly with high-dimensional and sparse data, the potential for joint models to provide deeper insights into disease progression and treatment effects is clear.

## Data Availability

No new data were created or analyzed in this study.

## Supplementary Materials

The following supporting information can be downloaded at: https://www.mdpi.com/article/10.3390/ijerph23040492/s1, PRISMA 2020 Checklist.

## Appendix A

**Table A1:** Methods of joint modeling studies with a univariate outcome for the longitudinal sub-model (N = 46).

Longitudinal Sub-Model	Semiparametric Survival Sub-Model						
	Cox PH ^a^	AFT ^b^	Cox PH with Piecewise Baseline Hazard	B-Spline	Frailty	Cox PH with Functional Predictors	Generalized Odds Rate Model
**Linear Mixed Effects**	[14,16]	[84] ^c^	[84] ^c^		[67,68,69,70,77,78]	[71]	
Robust linear mixed effects	[15]						
**Generalized Linear Mixed Effects**							
Logistic model				[29]			
Linear quantile mixed model	[18,19]						
Negative binomial	[22]						
Zero-inflated count model	[23,25]						
Copula gamma and Poisson	[27]						
Zero-inflated Beta model	[26]						
Two-part with logistic and log-normal regression	[28]						
**Generalized Linear Mixed Effects**							
Weighted generalized linear mixed effects	[53]						
**Latent Class Analysis**	[30,31]						
**Nonlinear Longitudinal Sub-Model**							
Splines	[34]	[35]				[33]	[37]
B-spline and LASSO						[38]	
Spatial–temporal multilevel model	[39]						
**Discrete-Time State Space Model**			[41]				
**Longitudinal Sub-Model**	**Parametric Survival Sub-Model**						
	**AFT**		**Competing Risk Model**	**Multistate Markov Model**		**Cox PH with Functional Predictors**	**Flexible Link Function**
**Linear Mixed Effects**	[73,75]		[76,78,80,81,84] ^c^	[83]			[77]
**Generalized Linear Mixed Effects**							
Linear quantile mixed model	[17]						
Ordinal logistic model			[21]	[20]			
Zero-inflated count model	[24]						
**Nonlinear Longitudinal Sub-Model**							
Splines						[36]	
Nonlinear mixed-effects model	[40]		[80]				
**Principal Component Analysis**: Functional Principal Component Analysis			[60]			[64]	

Note. ^a^. PH = proportional hazard. ^b^. AFT = accelerated failure time. ^c^. The study appeared more than once in the table due to multiple survival methods being adopted.

**Table A2:** Methods of joint modeling studies with a multivariate outcome for the longitudinal sub-model (N = 24).

Longitudinal Sub-Model	Survival Sub-Model			
	Non-Parametric	Semiparametric		Parametric
	Random Survival Forest	Cox PH ^a^	Frailty	AFT ^b^
**Linear Mixed Effects:** Multivariate normal		[42]	[43]	[74]
**Generalized Linear Mixed Effects**				
Continuous and binomial		[44]		
Continuous and ordinal				[46]
Linear quartile		[47]		
Two-regime model		[49]		
Piecewise mixed-effects model		[50]		
Skew-normal model		[45]		
Copula Gaussian and/or t		[52]		[51]
Bivariate binomial model				[48]
**Latent Class Analysis**		[32,54,55,56]		
**Latent Variables**		[57,58,59]		
**Principal Component Analysis:** Multivariate Functional Principal Component Analysis	[63]	[61,62]		
**Functional Mixed Model**		[65,66]		

Note. ^a^. PH = proportional hazard. ^b^. AFT = accelerated failure time.
